# Signal Integration in Plant Abiotic Stress Responses via Multistep Phosphorelay Signaling

**DOI:** 10.3389/fpls.2021.644823

**Published:** 2021-02-17

**Authors:** Jan Skalak, Katrina Leslie Nicolas, Radomira Vankova, Jan Hejatko

**Affiliations:** ^1^CEITEC - Central European Institute of Technology and National Centre for Biomolecular Research, Masaryk University, Brno, Czechia; ^2^Laboratory of Hormonal Regulations in Plants, Institute of Experimental Botany, Czech Academy of Sciences, Prague, Czechia

**Keywords:** multistep phosphorelay (MSP), cytokinin, ethylene, abscisic acid, light signaling, temperature, abiotic stress, Arabidopsis

## Abstract

Plants growing in any particular geographical location are exposed to variable and diverse environmental conditions throughout their lifespan. The multifactorial environmental pressure resulted into evolution of plant adaptation and survival strategies requiring ability to integrate multiple signals that combine to yield specific responses. These adaptive responses enable plants to maintain their growth and development while acquiring tolerance to a variety of environmental conditions. An essential signaling cascade that incorporates a wide range of exogenous as well as endogenous stimuli is multistep phosphorelay (MSP). MSP mediates the signaling of essential plant hormones that balance growth, development, and environmental adaptation. Nevertheless, the mechanisms by which specific signals are recognized by a commonly-occurring pathway are not yet clearly understood. Here we summarize our knowledge on the latest model of multistep phosphorelay signaling in plants and the molecular mechanisms underlying the integration of multiple inputs including both hormonal (cytokinins, ethylene and abscisic acid) and environmental (light and temperature) signals into a common pathway. We provide an overview of abiotic stress responses mediated via MSP signaling that are both hormone-dependent and independent. We highlight the mutual interactions of key players such as sensor kinases of various substrate specificities including their downstream targets. These constitute a tightly interconnected signaling network, enabling timely adaptation by the plant to an ever-changing environment. Finally, we propose possible future directions in stress-oriented research on MSP signaling and highlight its potential importance for targeted crop breeding.

## Prolog: Hormones and Stress Signal Transduction – the Chicken or the Egg?

Under natural conditions, plants are need to continuously maintain a balance between growth and defense or adaptive responses that are dictated by the severity, duration, and developmental timing at which any particular stress occurs (Claeys and Inzé, 2013). The essential question fundamental to understanding the mechanisms of hormonal control over any stress response is what is the very first action of the plant in response to (a sudden or long-term) change in environmental conditions? Does the stress response begin by activating the expression of defense/stress-responsive gene(s) that then subsequently impacts the hormonal status, or do plant hormones mediate the expression of stress-responsive transcription factors (TFs), or do hormonal signaling and metabolism act either synergistically with or independently on stress response gene expression?

The answer is not trivial, simply because stress responses employ mutually interconnected molecular cascades and networks, and a detailed description of these networks is outside the scope of this review. Recently available evidence suggests a combination of all the above response mechanisms. For instance, numerous studies have been published that show stress-controlled expression of key members of the MSP pathway, while genome-wide and proteomic studies of cytokinin (one of the key plant hormones and a major regulator of MSP activity, see below) action have revealed that cytokinin modulates levels of stress-related genes/proteins (Brenner et al., 2005, 2012; Bhargava et al., 2013; Černý et al., 2014; Brenner and Schmülling, 2015; Raines et al., 2016). On the other hand, the metabolism of stress-associated plant hormones abscisic acid (ABA) and cytokinins are also rapidly modulated by stress conditions. Stress initiation results in a rapid (under an hour) increase of ABA content but reduced endogenous levels of cytokinins together with attenuated expression of cytokinin receptor genes (Dobrá et al., 2015). Induction of jasmonic acid (JA) production was observed to occur within seconds after wounding (root excision), suggesting that in this case, regulation of endogenous hormonal levels is the primary stress response (Zhou et al., 2019). Down- and up-regulation of signaling components acting downstream of cytokinins and ABA, respectively, suggests a combined effect of hormone metabolism and signal transduction on the regulation of heat-stress responses (Skalák et al., 2016). In the case of ethylene, multiple environmental stresses stimulate its biosynthesis to act antagonistically with ABA in the regulation of shoot and root growth under water-limiting conditions (Sharp and Lenoble, 2002; Skirycz et al., 2011; Dubois et al., 2018). The expression of *ETHYLENE RESPONSE FACTOR 1* (*ERF1*) was gradually induced by drought stress treatment over 12 h (reaching a maximum after 1 h), and this plays a positive role in drought, salt, and heat stress tolerance via stress-specific gene regulation (Cheng et al., 2013).

All these findings (and many others described below) show how important plant hormone signaling (including the MSP) and metabolism are during early stress responses. In the next few sections, we attempt to summarize recent observations on the biological relevance of MSP-mediated stress adaptation strategies in plants (mostly in *Arabidopsis thaliana*).

## The Multistep Phosphorelay Cascade – Hormonal and Environmental Signal Transduction and Crosstalk

### The Cytokinin Signaling Pathway

**FIGURE 1:**
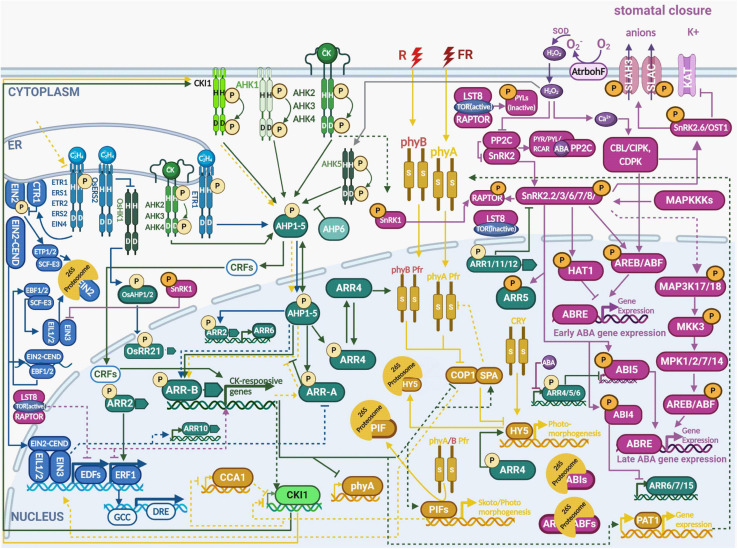
Multistep phosphorelay integrates hormonal and environmental signaling during abiotic stress responses. The output of multistep phosphorelay is affected by crosstalk with a number of individual signaling pathways. The crosstalk takes place either at the level of AHPs phosphorylation by the receptors with varying signal specificities or through interaction of the response regulators (both A-ARRs and B-ARRs) with various transcription factors [e.g., ELONGATED HYPOCOTYL 5 (HY5), CONSTITUTIVE PHOTOMORPHOGENIC 1 (COP1), ABSCISIC ACID RESPONSIVE ELEMENT-BINDING FACTORS (ABFs)], as well as SNF1-RELATED PROTEIN KINASE 2 (SnRK2) and phytochromes. See the main text for a detailed description of each signaling pathway. Color code: MSP signaling is depicted in green, ethylene signaling in blue, light signaling in ochre, abscisic acid in purple. The His/Asp phosphorylation is depicted by circled P in bright yellow, Ser/Thr/Tyr phosphorylation in orange. Created with BioRender.com.

Mainly due to historic reasons, much of the information about MSP has been on cytokinin signaling. Cytokinins are recognized by membrane-bound ARABIDOPSIS HISTIDINE KINASES (AHK2, AHK3, and AHK4/CRE1/WOL), located both at the plasma membrane and the endoplasmic reticulum (Caesar et al., 2011; Wulfetange et al., 2011). Recently, the quantitatively minor fraction of plasma membrane-located AHKs was shown to be functional in mediating the extracellular cytokinin signal (Antoniadi et al., 2020; Kubiasová et al., 2020). According to the current model (Figure 1), cytokinins bind to the CHASE domain (Hothorn et al., 2011) and activate intracellular histidine kinase (HK) activity possibly by changing receptor conformation; this then leads to sensor autophosphorylation (Hutchison and Kieber, 2002; Kieber and Schaller, 2018). The presence of phospho-His triggers downstream His-to-Asp-to-His-to-Asp phosphorelay. In the first (intramolecular) step, the phosphoryl group is passed from a conserved His residue of the HK domain to the conserved Asp residue located in the receiver domain of the receptor (Hwang and Sheen, 2001; Müller and Sheen, 2007). The small and mobile cytosolic proteins, ARABIDOPSIS HISTIDINE-CONTAINING PHOSPHOTRANSMITTERs (AHPs) 1-5, which shuttle between the cytosol and the nucleus, mediate the next step (Hwang and Sheen, 2001; Hutchison et al., 2006; Figure 1) and serve as a substrate for the phosphorylation of the terminal phosphate acceptors, the nuclear-located type-B ARABIDOPSIS RESPONSE REGULATORS (B-ARRs). Once the type B-ARRs are phosphorylated, inhibition on their GARP DNA-binding domain is released. This initiates the transcription of cytokinin-regulated genes, including the type-A *RESPONSE REGULATORS* (*A-ARRs*), the cytokinin primary-response genes, which also function as negative feedback regulators of the signaling pathway (Kieber and Schaller, 2018). In tandem with type-B ARRs, CYTOKININ RESPONSE FACTORS (CRFs) – closely related members of the Arabidopsis *APETALA 2/ETHYLENE RESPONSE FACTOR* (*AP2/ERF*) gene family, mediate a large fraction of the transcriptional response to cytokinins, affecting a set of cytokinin-responsive genes that largely overlaps with targets of type-B ARRs (Rashotte et al., 2006). For more details we would like to point the reader to some excellent comprehensive reviews, e.g., those by Zürcher and Müller (2016) or Kieber and Schaller (2018).

### Canonical Ethylene Signaling and Its Crosstalk With Multistep Phosphorelay

Ethylene is recognized by sensors belonging to subfamily 1, consisting of ETHYLENE RESPONSE 1 (ETR1) and ETHYLENE RESPONSE SENSOR 1 (ERS1) with a functional HK domain, and the HK-like sensor Ser/Thr kinases ETR2, ERS2, and ETHYLENE INSENSITIVE 4 (EIN4), that make up subfamily 2 (Etheridge et al., 2006). Ethylene binds to the hydrophobic pocket of the receptors via a copper cofactor, delivered there by the action of copper transporter, RESPONSE TO ANTAGONIST 1 (RAN1); (Schaller and Bleecker, 1995; Hirayama et al., 1999; Binder et al., 2010). Ethylene binding causes inactivation of both the receptor and the downstream Raf-like Ser/Thr protein kinase CONSTITUTIVE TRIPLE RESPONSE 1 (CTR1), a negative regulator of ethylene signaling (Kieber et al., 1993; Huang et al., 2003). In the absence of ethylene (in air), CTR1 phosphorylates and inhibits ETHYLENE INSENSITIVE 2 (EIN2), an ER membrane-localized Nramp homolog and a positive regulator of ethylene responses (Ju et al., 2012). That allows Skp1/Cullen/F-box (SCF) E3 ubiquitin ligase complexed with the EIN2-TARGETING PROTEIN 1 (ETP1) and ETP2 F-box proteins to target EIN2 for 26S proteasome-mediated degradation (Qiao et al., 2009). In parallel, the TFs and positive regulators of ethylene-induced gene expression EIN3, ETHYLENE-INSENSITIVE3-LIKE 1 (EIL1) and EIL2 are targeted for ubiquitination by an EBF1- and EBF2- (F-box proteins) containing SCF E3 complex (Guo and Ecker, 2003; Potuschak et al., 2003; Gagne et al., 2004; Binder et al., 2007; An et al., 2010). The ethylene-induced inactivation of all ethylene sensors leads to the attenuation of CTR1 kinase activity, dephosphorylation of EIN2 and cleavage of its C-terminus (EIN2-CEND). In the cytoplasm, EIN2-CEND inhibits translation of EBF1 and EBF2 by targeting their mRNAs to the processing bodies and subsequent degradation (Li et al., 2015; Merchante et al., 2015). This then upregulates the levels of their targets EIN3, EIL1 and EIL2. EIN2-CEND is also translocated to the nucleus (Qiao et al., 2012; Wen et al., 2012), where it recognizes EIN2 NUCLEAR ASSOCIATED PROTEIN 1 (ENAP1) and promotes EIN3 binding to specific (ENAP1-recognized) chromatin regions by upregulating histone acetylation (Zhang et al., 2017). Specific histone posttranslational modifications seem to be important for ethylene-regulated gene expression (Binder, 2020; and references therein). EIN2-CEND-stabilized EIN3, EIL1 and EIL2 upregulate the expression of ethylene induced genes, including the TFs ETHYLENE RESPONSE DNA BINDING FACTOR 1 (EDF1), EDF2, EDF3 and EDF4 and ETHYLENE RESPONSE FACTORs (ERF)s. Activated ERFs initiate a transcriptional cascade leading to the activation of many ethylene-responsive genes by binding to specific *cis*-acting GCC box and DRE elements depending on whether the stress conditions are abiotic or biotic (reviewed in Müller and Munné-Bosch, 2015). For more detailed information about the ethylene signaling pathway, see the recent review by Binder (2020).

The fact that two ethylene sensors belong to HKs indicates possible signaling crosstalk with the MSP pathway. However, ethylene sensors from both subfamilies mediate ethylene signal transduction primarily through the CTR1/EIN2/EIN3 pathway and it took nearly two decades until evidence pointing to intense cytokinin/ethylene signaling crosstalk was revealed (Figure 1). ETR1 (containing a receiver domain) was shown to interact with AHP1, AHP2, AHP3 and AHP5 (Urao et al., 2000; Scharein et al., 2008; Zdarska et al., 2019). ETR1-dependent phosphorylation of type-B ARR2 and activation of the type-A ARR6 promoter was demonstrated in Arabidopsis protoplasts (Hass et al., 2004; Cho and Yoo, 2007). The *arr2* mutant is less sensitive to treatment with the ethylene precursor 1-aminocyclopropane-1-carboxylic acid (ACC) probably due to a positive effect of ARR2 on the expression of *ERF1*, an ethylene-responsive member of the ERF/AP2 transcription factor family (Hass et al., 2004). A recent study showed that ETR1-mediated ethylene signaling controls RAM size via ARR3 independently of EIN2 and in a distinct way from canonical CTR1/EIN2/EIN3 signaling (Street et al., 2015; Zdarska et al., 2019). Importantly, canonical ethylene signaling seems to control the sensitivity of MSP to cytokinins, possibly via ethylene-regulated expression of type-B *ARR10* (Zdarska et al., 2019). In rice, the ethylene receptor OsERS2 physically interacts with cytosolic HK MHZ1/OsHK1 (homolog of Arabidopsis AHK5, see below) through its GAF domain in an ethylene-dependent manner and this interaction inhibits MHZ1/OsHK1 kinase activity. OsHK1 phosphorylates the response regulator OsRR21 via the phosphotransfer proteins OsAHP1 and OsAHP2, which are both required for ethylene-mediated root-growth inhibition (Zhao et al., 2020). Considering the strong regulation of ethylene biosynthesis by cytokinins (reviewed in Zdarska et al., 2015) MSP output must be considered as a combined output of both cytokinin and ethylene signals.

### Crosstalk Between Light Signaling and Multistep Phosphorelay

Light is an essential environmental factor influencing plant development and adaptive responses. Photochemically active light receptors such as phytochromes, cryptochromes, phototropins, ZEITLUPE (ZTL) and the UV-B RESISTANCE 8 (UVR8) family proteins affect plant behavior in response to both quantity and quality of light. The individual signaling branches mediating responses to a wide range of wavelengths as well as their interplay has been extensively studied (e.g., Quail et al., 1995; Rockwell et al., 2006; Li et al., 2011; Wang Q. et al., 2018). Since light signaling is a very broad topic, we focus here only on the direct interaction of phytochrome-mediated light signaling components with MSP.

Phytochromes are light-regulated Ser/Thr kinases, mediating plant response to red (R) and far-red (FR) light. Phytochromes are synthesized as light-insensitive apoproteins and the functional, i.e., light photoswitchable holoproteins (phys) are assembled following binding with the light-sensitive, linear tetrapyrrole chromophore cofactor phytochromobilin, that is imported to the cytosol from plastids (Terry et al., 2002; Wang, 2015). Upon absorbing R-light the phytochromes switch from the inactive (R-light absorbing) Pr form to the active (FR-light absorbing) Pfr form. The only phy activated by FR (thanks to partial overlap between Pr and Pfr absorption spectra) in Arabidopsis is phyA (Casal et al., 2014; Ballaré and Pierik, 2017). Activated phys translocate to the nucleus either alone [as for phytochrome B (phyB)] or complexed to phy-transporting proteins (as in the case of phyA). The nuclear-located phys control light-responsive genes through two main routes. The first is based on phosphorylating PHY-INTERACTING FACTORs (PIFs), thus targeting them for subsequent degradation via the CUL4^*COP1–SPA E3*^ ubiquitin ligase (Zhu et al., 2015). PIFs control both skotomorphogenesis (by activating genes expressed during the dark phase) and photomorphogenesis (by inhibiting light-induced genes). The second pathway is controlled by phytochrome-mediated inhibition of transcriptional repressors CONSTITUTIVE PHOTOMORPHOGENIC (COP), DE-ETIOLATED (DET), and FUSCA (FUS; Huang et al., 2014). COP1 interacts with SUPPRESSOR OF PHYA (SPA) proteins, forming an E3 ubiquitin ligase complex that targets light-responsive TFs for 26S proteasome-mediated degradation. Activated phys inhibit the COP1-SPA, allowing accumulation of photomorphogenesis-promoting TFs including ELONGATED HYPOCOTYL 5 (HY5) (Huang et al., 2014; Lu et al., 2015; Sheerin et al., 2015; Figure 1). More details on the complex issue of phytochrome signaling and its interaction with hormonal signaling can be found in excellent reviews by Wang (2015); Xu et al. (2015) and De Wit et al. (2016).

One of the first pieces of evidence for direct crosstalk between MSP and light signaling was presented by Sweere et al. (2001). ARR4, a type-A ARR, interacts with phyB and stabilizes its active Pfr form, preventing dark reversion to the inactive Pr form, thus making plants more sensitive to red light (Sweere et al., 2001). ARR4 action on phyB and ARR4-controlled regulation of photomorphogenesis is dependent on its conserved phosphorylatable Asp 95, and this ARR4-mediated stabilization of phyB is reversed in the presence of cytokinins, providing a direct functional link between MSP and light signaling (Mira-Rodado et al., 2007).

Expression of the constitutively active HK *CKI1* was found to be under the control of phyA, probably via PHY-INTERACTING FACTOR 1 (PIF1) and CIRCADIAN CLOCK ASSOCIATED 1 (CCA1). Plants defective in phyA and HEME OXYGENASE1 (HO1), whose product is involved in the production of phytochromobilin, show misregulation of *CKI1* expression, associated with attenuation of MSP responsiveness to cytokinins and occurrence of cytokinin-related phenotypes (Dobisova et al., 2017). Light-controlled phyA-dependent transcriptional regulation of *CKI1* expression is a conceptually novel signaling mechanism in plants. Here, an environmental regulatory signal (light) controls the activity of (MSP) signaling pathway by transcriptional regulation of the constitutively active HK, thus controlling MSP sensitivity to its primary signal, cytokinin (Dobisova et al., 2017). On the other hand, exogenous cytokinins were reported to negatively modulate phyA expression (Cotton et al., 1990; Brenner et al., 2012). The negative impact of cytokinins on phyA transcription is fine-tuned by up-regulating both PHYTOCHROME A SIGNAL TRANSDUCTION 1 (PAT1), a positive regulator of phyA signaling and SPA1, its negative regulator (Brenner et al., 2005, 2012). Furthermore, cytokinins suppress the function of the E3 ubiquitin-protein ligase COP1, which mediates ubiquitination and subsequent proteasomal degradation of HY5, associated with repression of photomorphogenesis in darkness (Srivastava et al., 2015) and increased accumulation of the bHLH family proteins PIFs and EIN3 (Alabadí and Blázquez, 2008; Zhong et al., 2009; Figure 1). HY5 acts as a downstream intermediate in the CRYPTOCHROME 1 (CRY1; photoreceptor absorbing blue light) signaling pathway (Vandenbussche et al., 2007). Both the cytokinin (via ARR4) and CRY pathways upregulate HY5 protein levels that consequently regulates the transcription of many genes by binding directly to their *cis*-regulatory elements. Thus, regulation of the stability of HY5 protein has been suggested to be a point of convergence between both signaling pathways (Shin et al., 2007, 2013; Vandenbussche et al., 2007; Gangappa and Botto, 2016).

Apart from the mutual light-MSP interactions, a positive role was shown for light in ethylene signaling, as well as light-dependent stimulation of ETR1 and EIN4 expression. On the other hand, *ETR2* and *ERS2* gene expression was attenuated during the exposure to light (Grefen et al., 2008). We saw earlier how the ethylene receptor (ETR1) and downstream MSP components (AHP1, AHP2, AHP3, and AHP5) (Hass et al., 2004; Scharein et al., 2008; Street et al., 2015; Zdarska et al., 2019; Zhao et al., 2020) are linked. Thus, the light-controlled expression of ethylene sensor-encoding genes might represent another way of MSP-mediated integration of light with plant hormone signaling pathways.

### Histidine Kinases Not Responsive to Hormone Signals

In *Arabidopsis thaliana*, several histidine kinases have been identified, namely AHK1, AHK5, and CYTOKININ-INDEPENDENT 1 (CKI1) that do not mediate hormone responses (Figure 1). The sensory histidine kinase AHK1 was previously shown to transduce the external osmolarity signal (Urao et al., 1999; Tran et al., 2007) and its exact role in cytokinin signaling has not been elucidated. CKI1 was identified by Kakimoto (1996) using activation mutagenesis, and was proposed to act as a cytokinin sensor. Later on, however, CKI1 was found to be a constitutively-active HK, activating MSP signaling in a cytokinin-independent fashion (Hwang and Sheen, 2001; Yamada et al., 2001; Hejátko et al., 2009), and controlling many aspects of plant development including root growth, vascular tissue formation in the inflorescence stem and female gametophyte development (Hwang and Sheen, 2001; Pischke et al., 2002; Hejátko et al., 2003, 2009; Deng et al., 2010; Yuan et al., 2016; Liu et al., 2017). AHK5 has been shown to play a key role in integrating multiple signals in guard cells – independent of ABA signaling via H_2_O_2_ homeostasis – possibly acting as redox sensor (Desikan et al., 2008). In terms of expression patterns, *CKI1* is expressed in female gametophytes, developing seeds, shoot apical meristem, shoot and root vasculature and the lateral root cap, whereas *AHK1* transcript levels are most abundant in roots and is transcriptionally regulated by osmotic stress (Urao et al., 1999; Pischke et al., 2002; Hejátko et al., 2003, 2009; Dobisova et al., 2017). The AHK5 transcript was present in roots, flowers and siliques, and at low levels in leaves, where it plays a unique role in stomatal signaling (Desikan et al., 2008; Horak et al., 2011). Several researchers have shown that these non-hormone responsive HKs are necessary for modulating the plant responses to stress, and is described in detail below in Section “Sensing Abiotic Stresses via Multistep Phosphorelay.”

### Canonical ABA Signaling

Abscisic acid has been known for decades as an essential plant hormone for plant development and stress responses; for instance, ABA is elevated when plants suffer from decreased water potential, as well as during seed maturation and fruit ripening (Wright, 1977; Rodríguez-Gacio Mdel et al., 2009). The ABA signaling network includes the receptors PYRABACTIN RESISTANCE 1 (PYR1)/PYR1-LIKE (PYLs)/REGULATORY COMPONENTS OF ABA RECEPTORS (RCARs), which, upon ABA binding, form a complex with TYPE 2C PROTEIN PHOSPHATASES (PP2Cs), resulting in the PP2C inactivation (Ma et al., 2009; Park et al., 2009). This, in turn, releases members of SNF1-RELATED PROTEIN KINASEs 2 (SnRK2s) subfamily (see below), which get activated by autophosphorylation (Belin et al., 2006) and further phosphorylate several transcription factors such as ABA INSENSITIVE 4 (ABI4), ABI5, ABA-RESPONSIVE ELEMENT BINDING FACTORS (ABFs) as well as membrane ion channel proteins like SLOW ANION CHANNEL-ASSOCIATED1 (SLAC1) and potassium channel protein 3-KETO-ACYL-COA THIOLASE 1 (KAT-1) as well as MITOGEN-ACTIVATED PROTEIN KINASE1 (MPK1) and MPK2 (reviewed in Fujita et al., 2013; Yu et al., 2016; Dejonghe et al., 2018) (Figure 1). Based on their ability to be activated by ABA, SnRK2s could be divided into three subclasses: Subclass I – not responsive to ABA (SnRK2.1, SnRK2.4, SnRK2.5, SnRK2.9, and SnRK2.10), Subclass II - weakly activated by ABA (SnRK2.7 and SnRK2.8) and Subclass III - strongly activated by ABA (SnRK2.2, SnRK2.3, and SnRK2.6) (Ng et al., 2014). Subclass III kinases are essential components of ABA signaling pathway, as indicated by impaired ABA responses in triple mutants lacking SnRK2.2, SnRK2.3, and SnRK2.6 (Fujii and Zhu, 2009; Fujita et al., 2009; Nakashima et al., 2009).

Another regulatory circuit affecting SnRK activity employs TARGET OF RAPAMYCIN (TOR) kinase (Wang P. et al., 2018; Belda-Palazón et al., 2020). Under unstressed (growth favorable) conditions, TOR signaling complex represses ABA-mediated stress signaling via phosphorylation of PYL receptors. This phosphorylation leads to SnRK2 kinase inactivation, which associates with disruption of PYL and PP2C phosphatase effector function, thus promoting plant growth. In the presence of stress, ABA signaling represses function of TOR complex via phosphorylation of RAPTOR, the regulatory subunit of TOR, leading to stress responses and growth inhibition (Wang P. et al., 2018). Interestingly, the growth promoting role has been attributed to SnRK2s, too. In the absence of ABA, SnRK2s-containing complexes recognize SnRK1, a negative regulator of TOR. That allows TOR activation and growth under non-stress (growth-promoting) conditions (Belda-Palazón et al., 2020). SnRK1, via its α subunit, has been shown to interact and phosphorylate RAPTOR1B, one of two *Arabidopsis* RAPTOR/KOG1 homologs, which seems to control energy-demanding processes including translation. Surprisingly, SnRK1 might also be involved in regulating photosynthesis via phosphorylaton of chloroplast proteins (Nukarinen et al., 2016). The ability of SnRK1 to act upstream of TOR has been also proposed in stress- (nutrient or energy deficiency) induced autophagy (Soto-Burgos and Bassham, 2017).

The ABA-signaling pathway is an essential regulator of stomatal movement which is either dependent or independent on Ca^2+^ levels (McAinsh and Hetherington, 1998; Webb et al., 2001; Siegel et al., 2009). Previously was reported that increases in Ca^2+^ are the components of the guard cell ABA-nuclear as well as ABA-turgor signaling pathway (Webb et al., 2001). However, part of Ca^2+^ signaling components are found in the guard cells to act as a separate Ca^2+^-independent ABA signaling pathway (Allan et al., 1994; Blatt and Grabov, 1997; Webb et al., 2001). Ca^2+^ elevation can accelerate the stomata closure by enhancing plasma membrane SLOW ANION CHANNEL-ASSOCIATED 1 (SLAC1) and cytoskeletal rearrangement (Waidyarathne and Samarasinghe, 2018). Nevertheless, Ca^2+^ increase seems not to be the only essential component of this process, thus the exact role of Ca^2+^ in ABA signaling needs to be explained (Waidyarathne and Samarasinghe, 2018).

Similarly to cytokinin and ethylene, ABA responses also include chromatin-mediated control of gene expression by regulation of chromatin-remodeling complexes by, for instance, the SWI/SNF subgroup complexes forming around the chromatin-remodeling ATPase BRAHMA (BRM) (Chinnusamy and Zhu, 2009; Yaish et al., 2011; Han and Wagner, 2014; Han et al., 2015). SnRK2s and PP2Cs directly target conserved phosphorylation site in the C-terminal region of BRM (Peirats-Llobet et al., 2016). Whereas PP2CA dephosphorylates BRM, likely as a repressive mechanism of BRM-dependent modulation of ABA responses, SnRK2s phosphorylate BRM to release BRM-mediated repression of *ABI5* expression (Peirats-Llobet et al., 2016).

Since ABA interacts tightly with MSP signaling during the abiotic stress response, the mutual crosstalk might occur at several levels including regulation of phosphorylation of individual signaling components as well as regulation of dynamic chromatin state (Chinnusamy and Zhu, 2009; Yaish et al., 2011; Han and Wagner, 2014). These interactions are described further in the text.

Altogether, the MSP pathway is not just the cytokinin signaling transduction system, but it acts as a signaling hub, integrating both intrinsic (mostly hormonal) regulation with that from environmental signals, including light, osmoregulation, and temperature (Figure 1). The importance of integration of cytokinin, ethylene, ABA, and light signaling pathways is what underlies the ability of plants to display such plasticity in continual adjustment to the changing environment.

## Sensing Abiotic Stresses via Multistep Phosphorelay

**FIGURE 2:**
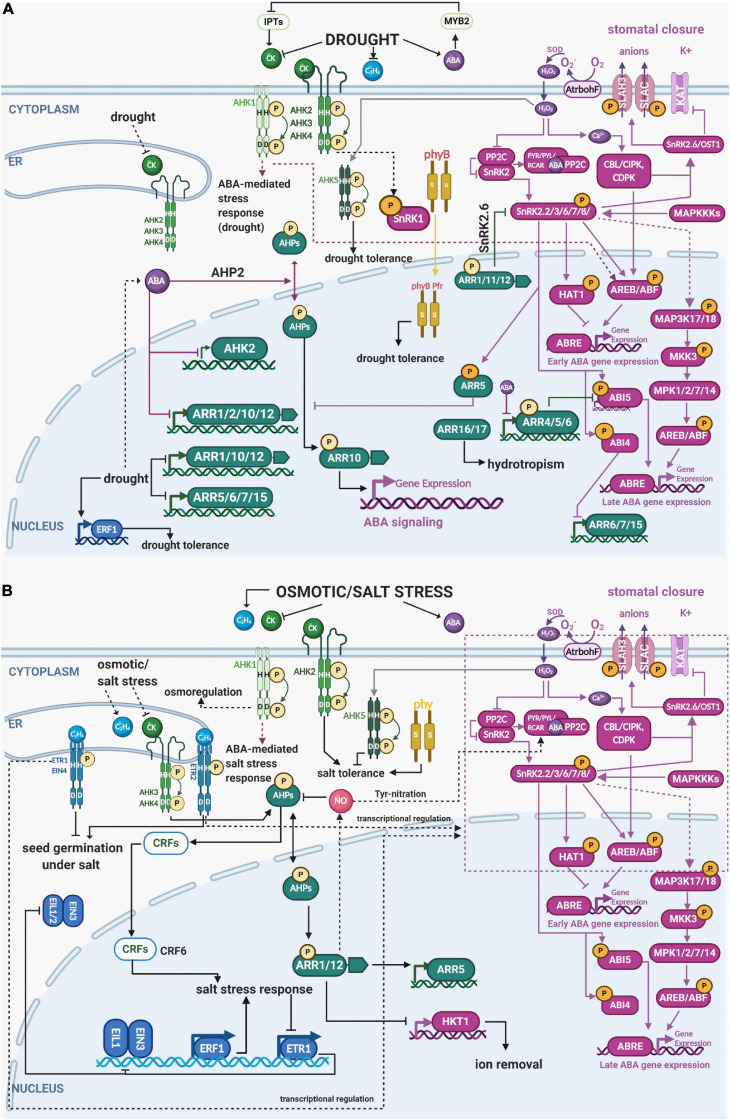
Integration of the different components of ethylene, cytokinin, ABA and light signaling in multistep phosphorelay during drought and osmotic/salt stress. **(A)** Drought stress stimulates ABA and ethylene content while reducing the endogenous levels of cytokinins. Both cytokinin-responsive HKs AHK2, AHK3, and AHK4 as well as cytokinin-independent AHK1 and AHK5 contribute to the drought-regulated MSP activity. Strong crosstalk (mostly mutually negative) occurs between MSP and canonical ABA signaling. Ethylene response factors (ERFs) are also induced and play mostly positive role in stress responses. **(B)** Like drought, osmotic/salt stress also stimulates ABA and ethylene production, thus activating ABA and ethylene signaling components, while downregulating cytokinin levels and the expression of its downstream signaling components. Also, expression of *ETR1* is reduced, enabling the continued activity and participation of downstream components of ethylene signaling (EIL1, EIN3, and ERFs) in the activation of different response factors necessary for the osmotic/salt stress response. Phytochromes are also implicated to be involved in drought and osmotic/salt stress responses, particularly phyB which was shown to enhance osmotic and drought tolerance. See the main text for a detailed description of each signaling pathway and their crosstalk in response to stress. Color code: MSP signaling is depicted in green, ethylene signaling in blue, light signaling in ochre, abscisic acid in purple. The His/Asp phosphorylation is depicted by circled P in bright yellow, Ser/Thr/Tyr phosphorylation in orange. Created with BioRender.com.

Stress, in general, affects the metabolism of cytokinins, ABA, and ethylene, which in turn interact with specific kinases to regulate many stress-related biological processes ranging from regulation of shoot growth under stress to stomata movement. Drought results in the reduction of cytokinin content by modulating cytokinin metabolism and stimulating ABA and ethylene contents in a wide range of terrestrial plants (e.g., Dobra et al., 2010; Guo and Gan, 2011; Nishiyama et al., 2011; Macková et al., 2013; Todaka et al., 2017; Figure 2). Even though there is a correlation between hormone metabolism and MSP activity, a significant set of stress responses take place independently of the hormonal pool (O’Brien and Benková, 2013). In this review, we focus on the role of individual MSP components and molecular links in pathways for abiotic stress responses (Table 1). Detailed descriptions of the stress-related changes in hormonal metabolism are left out as they are beyond the scope of this review.

**TABLE 1:** Role of MSP components from *Arabidopsis thaliana* L. in various environmental stimuli.

***Gene ID***	**AGI code**	**Description**	**Regulatory function in stress response**	**Type of stress**	**Regulatory function in ABA response**	**References**
*AHK1*	AT2G17820	Histidine kinase	Positive	Drought, salt	Positive	Tran et al., 2007
*AHK2*	AT5G35750	Cytokinin receptor kinase	Negative	Cold, drought, salt	Negative	Tran et al., 2007; Jeon et al., 2010
*AHK3*	AT1G27320	Cytokinin receptor kinase	Negative	Cold, drought, salt	Negative	Tran et al., 2007; Jeon et al., 2010
*AHK4/CRE1*	AT2G01830	Cytokinin receptor kinase	Negative	Salt, drought	Negative	Tran et al., 2007
*AHK5*	AT5G10720	Histidine kinase	Negative	Drought, salt		Desikan et al., 2008; Pham and Desikan, 2012
*ARR1*	AT3G16857	Transcription factor (Type B-ARRs)	Positive	Cold	Unknown	Jeon and Kim, 2013
*ARR1, ARR12*	AT3G16857, AT2G25180	Transcription factors (Type B-ARRs)	Negative	Salt	Unknown	Mason et al., 2010
*ARR1, ARR10, ARR12*	AT3G16857 AT4G31920 AT2G25180	Transcription factors (Type B-ARRs)	Negative	Drought	Negative	Nguyen et al., 2016
*ARR5*	AT3G48100	Primary response genes (Type A-ARRs)	Negative Positive	Drought, salt	Positive Positive	[Bibr B78][Bibr B126]
*ARR7*	AT1G19050	Primary response genes (Type A-ARRs)	Negative	Cold, drought	Negative	Jeon et al., 2010
*ARR5*	AT3G48100	Primary response genes (Type A-ARRs)	Negative	Cold	None	Jeon et al., 2010
*ARR6*	AT5G62920	Primary response genes (Type A-ARRs)	Negative	Cold	Negative	Jeon et al., 2010
*ARR7*	AT1G19050	Primary response genes (Type A-ARRs)	Negative	Cold	Negative	Jeon et al., 2010
*ARR16 ARR17*	AT2G40670 AT3G56380	Primary response genes (Type A-ARRs)	Positive	Root hydrotropism	–	Chang et al., 2019

### Hormonal Crosstalk in Drought, Osmotic and Cold Stress

Abscisic acid mediates one of the fastest plant adaptations to drought, i.e., stomata closure to control the trade-off between CO_2_ uptake and water loss by transpiration (Sah et al., 2016; Vishwakarma et al., 2017). However, ABA controls also mid- and long-term plant adaptations to abiotic stresses including control of plant architecture, wherein ABA tightly interacts with cytokinins. The current model predicts that cytokinin-regulated MSP attenuates the expression of ABA-inducible genes involved in the stress response. Stress-induced ABA accumulation in turn downregulates cytokinin biosynthesis via the MYB2 TF, relieving the repression on MSP and activation of ABA- and stress-inducible genes (Li et al., 2016). ABA-mediated inhibition of cytokinin signaling initiates reshaping of the plant body by downregulating growth in the shoot but accelerating root growth. That allows the plant to diminish water loss while increasing water uptake from deeper layers of the soil (Li et al., 2016). In line with that, MSP signaling mutants, including mutants defective in cytokinin sensors AHK2, AHK3, and AHK4 and type-B ARRs ARR1, ARR10, and ARR16 are hypersensitive to ABA and show higher drought resistance (Tran et al., 2007, 2010; Nguyen et al., 2016). Both ABA and drought were shown to downregulate expression of *ARR1, ARR10*, and *ARR12* (Nguyen et al., 2016). ABA also downregulates *ARR2* but not *AHK3* or *AHK4* (Takatsuka and Umeda, 2019), and a possible role for ABA in the control of nucleocytoplasmic partitioning of AHP2 was reported (Marchadier and Hetherington, 2014). Interestingly, the osmosensor AHK1 is not a negative, but rather a positive regulator of the ABA-mediated stress response (Tran et al., 2007), suggesting certain specificity at the level of signals initiating MSP-regulated (drought) stress response (Hai et al., 2020).

The negative interaction between ABA and MSP activity is mediated not only at the level of ABA-controlled downregulation of cytokinin biosynthesis, but also at the level of interaction between components of canonical ABA signaling and MSP. ABA-activated ABI4 binds the promoters and downregulates *ARR6, ARR7*, and *ARR15*; single and multiple mutant lines *arr4*, *arr6, arr7*, and *arr15* were shown to be hypersensitive to ABA (Jeon et al., 2010; Wang et al., 2011; Huang et al., 2017). SnRK2.2, SnRK2.3, and SnRK2.6 phosphorylate several Ser residues of ARR5, the type-A ARR and negative regulator of MSP signaling. This leads to stabilization of the ARR5 protein, thus enhancing ABA response and drought tolerance by suppressing the cytokinin signaling (Huang et al., 2018; Figure 2). On the other hand, the phosphoproteomic study performed by Dautel et al. (2016) proposed an AHK2/AHK3-dependent phosphorylation of Thr6 and Tyr19 of KIN10, one of the two subunits of SnRK1, acting in energy stress (Baena-González and Sheen, 2008). SnRK1 down-regulation has been previously linked to cytokinin and auxin signaling based on global gene regulation by KIN10 (Radchuk et al., 2006; Baena-González et al., 2007), whereas ethylene signaling was negatively regulated by SnRK1 phosphorylation-mediated inactivation of EIN3 (Kim et al., 2017) leading to a growth-defense trade-offs model via cell cycle regulations (Eichmann and Schäfer, 2015; Filipe et al., 2018). The negative relationship between ABA and cytokinin levels/signaling is bi-directional. Upregulation of cytokinin biosynthesis via upregulation of *AtIPT8* resulted in ABA insensitivity in seed germination (Wang et al., 2011). Furthermore, when endogenous cytokinins are elevated, ABA was unable to downregulate genes for type-A ARRs ARR4, ARR5, and ARR6 that physically interact with ABI5 and downregulate *ABI5* expression (Wang et al., 2011). The lower sensitivity to ABA under high endogenous cytokinin levels is likely mediated by the cytokinin-responsive type-B ARRs ARR1, ARR11, and ARR12 that physically interact with SnRK2s and repress the kinase activity of SnRK2.6 (Huang et al., 2018). At the transcriptional level, the cytokinin-dependent regulation of ABA signaling might be mediated through ARR10, which was found to bind promoters of numerous members of ABA signaling (Zubo et al., 2017). Since canonical ABA signaling stimulates the expression of many transcription factors essential for stress responses and seed development (reviewed in Nakashima and Yamaguchi-Shinozaki, 2013; Kumar et al., 2019), the interaction with MSP components contributes to fine-tuning of the final stress response and regulates developmental processes (Kieber et al., 1993; Clark et al., 1998; Ju et al., 2012).

In the response to salt stress, cytokinins act through ARR1 and ARR12 to repress expression of the Arabidopsis HIGH-AFFINITY K^+^ TRANSPORTER 1;1 (AtHKT1;1) which is responsible for removing sodium ions from root xylem. Cytokinins were also shown to regulate salt stress−induced expression of the type−A response regulator *ARR5* predominantly via ARR1 and ARR12, revealing the action of particular MSP components in the roots to control sodium accumulation in the shoots (Mason et al., 2010).

**FIGURE 3:**
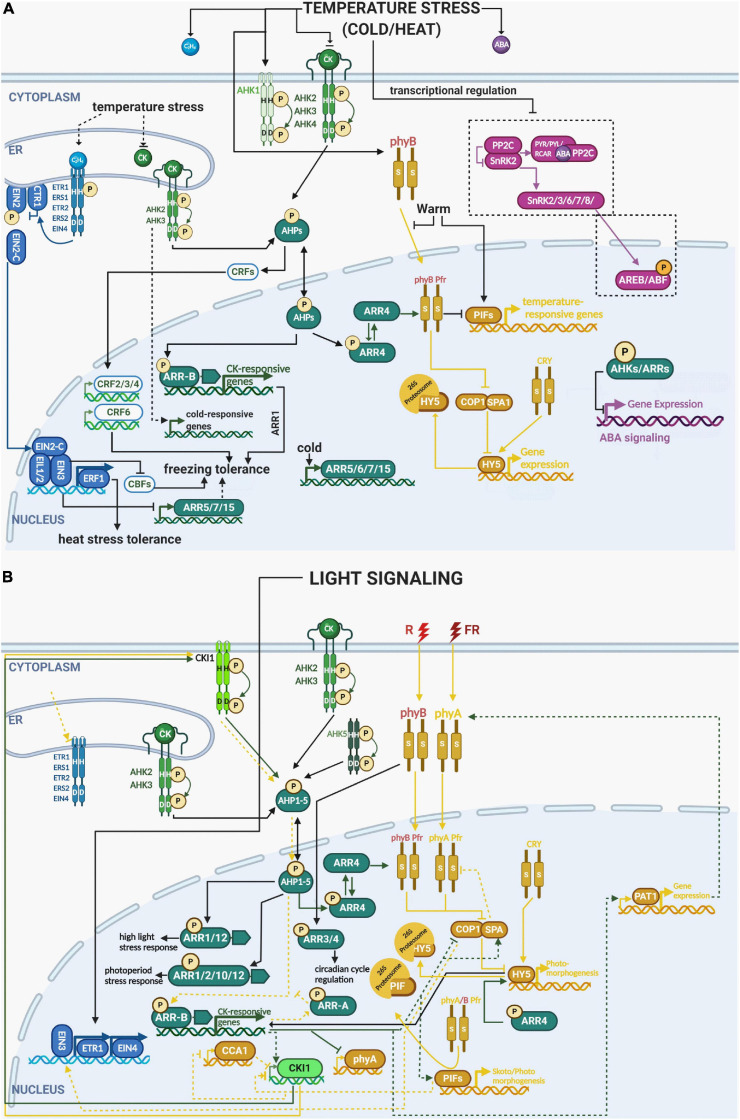
Integration of the different components of ethylene, cytokinin, ABA and light signaling pathways in multistep phosphorelay in temperature and light responses. **(A)** Heat stress: similarly to drought and osmotic stress, heat stress also enhances ABA and ethylene levels while downregulating cytokinin levels and the expression of cytokinin receptors (AHK2, 3, 4). ETR1 and ERF1 play a positive role in heat tolerance; however, downstream components of ABA (PYR/PYL/RCAR, PP2Cs, SnRK2s, and ABFs) are downregulated. **(B)** Cold stress: low temperature reduces the levels of cytokinin, but activates cytokinin receptors (AHKs), putatively in a cytokinin-independent manner. Several downstream components of the cytokinin signaling pathway are also activated (AHP2,3,5, B-ARR 1, A-ARR 5, 6, 7, 15) by cold temperature and correlated with enhanced cold tolerance in *Arabidopsis thaliana* (modified from Zdarska et al., 2015). phyB was demonstrated to be a temperature sensor, which interacts with ARR4, the key regulator providing a molecular link between MSP and light (and possibly temperature) signaling. Light response: light can also attenuate the levels of ethylene. ETR1 and EIN4 expression are dependent on light stimulation. ETR1 also genetically interacts with both phyA and phyB that govern germination and the growth response to various light regimes. Cytokinins were also shown to maintain photoprotective mechanisms during high light stress, mediated by AHK2 and AHK3. See the main text for a detailed description of each signaling pathway and their crosstalk in response to stress. Color code: MSP signaling is depicted in green, ethylene signaling in blue, light signaling in ochre, abscisic acid in purple. The His/Asp phosphorylation is depicted by circled P in bright yellow, Ser/Thr/Tyr phosphorylation in orange. Created with BioRender.com.

Interestingly, cold transiently stimulates the expression of *ARR5, ARR6, ARR7*, and *ARR15* similarly to dehydration (Jeon et al., 2010; Kang et al., 2012), most probably to attenuate cytokinin signal transduction and suppress growth. Shi et al. (2012) found that ethylene biosynthesis and signaling negatively regulates the freezing stress response in *Arabidopsis* by repressing cold-inducible C-REPEAT BINDING FACTORs (CBFs) and the type-A ARR genes *ARR5, ARR7*, and *ARR15*. This ethylene-mediated repression was supposed to be mediated by direct binding of EIN3 to the promoters of type-A ARRs, thus potentially representing another mechanistic link between canonical ethylene signaling and MSP during plant desiccation (Figures 2, 3).

### Hormone-Independent Abiotic Stress Responses

AHK1 gain-of-function mutations revealed that the osmo-responsive AHK1 was a positive regulator of drought and salt stress responses acting upstream of ABA signaling (Tran et al., 2007) (Figure 2). Disturbed up-regulation in the *ahk1* mutant of many stress- or ABA-inducible genes, including ABSCISIC ACID RESPONSIVE ELEMENTS-BINDING PROTEIN 1 (AREB1), NAC DOMAIN CONTAINING PROTEIN (ANAC), and DRE-BINDING PROTEIN 2A (DREB2A) transcription factors and their downstream genes may explain the increased stress tolerance of *AHK1* overexpressing plants (Tran et al., 2007). Moreover, AHK1 was shown to control stomatal density as well as the transcription of several stress-responsive genes. *ahk1* mutants have a higher transpiration rate and reduced ABA sensitivity, resulting in higher susceptibility to uncontrolled soil drying (Kumar et al., 2013).

Another experiment showed attenuated expression of *AHK2* in the first hours after exposure to reduced water potential (Figure 2; Kumar and Verslues, 2015). The examination of *ahk2-2* mutant lines showed hypersensitive response to salt stress, while root elongation in *ahk3-3* mutants was increased following transfer to low water potential media when compared to wild type (Kumar and Verslues, 2015). These results show the specific role of individual HKs in osmotic stress acclimation and provide evidence for the specificity of individual MSP components to different types of stress.

AHK5 was shown to mediate ABA-independent stomata closure in response to H_2_O_2_ produced by the NADPH oxidase AtrbohF (Desikan et al., 2006), which is induced by abiotic stimuli including darkness, nitric oxide (NO), and ethylene (Desikan et al., 2008). AHK5 inhibits root elongation by negatively regulating the ABA and ethylene signaling pathways (Iwama et al., 2007). On the other hand, AHK5 was also demonstrated to be a negative regulator of salinity tolerance, while providing plants with drought tolerance by promoting ABA-independent stomatal closure by maintaining H_2_O_2_ homeostasis in guard cells (Desikan et al., 2008; Pham and Desikan, 2012; Figure 2). Moreover, AHK5 is required for full immunity toward the necrotrophic fungus *Botrytis cinerea* and the virulent bacterium *Pseudomonas syringae* pv. tomato DC3000. It thus acts as an integrator of both abiotic and biotic stress responses in *Arabidopsis* (Pham and Desikan, 2012; Pham et al., 2012).

Nitric oxide has been recently characterized as a plant growth regulator involved in the control of many important developmental and adaptive responses, including the abiotic stress response (reviewed e.g., in Sami et al., 2018). Cytokinins induce NO production via MSP signaling, and cytokinin-induced NO contributes substantially toward plant morphological responses to cytokinin (Tun et al., 2008). Importantly, NO was shown to *S*-nitrosylate the conserved Cys 115 of AHP1, thus impairing its ability to mediate phosphotransfer to ARR1. This is an example of a mechanism by which the redox signal is integrated into cytokinin signaling – highlighting the complex coordination underlying plant growth and development (Feng et al., 2013). NO seems to antagonize not only cytokinin, but also ABA signaling. NO biosynthetic mutant *nia1 nia2 noa1-2* is hypersensitive to ABA, leading to highly efficient ABA-mediated control over stomata closure (Lozano-Juste and León, 2010a) and NO production under growth-favorable conditions represses inhibition of seed developmental transitions by ABA (Lozano-Juste and León, 2010b). Increase in NO levels post-translationally modifies members of the ABA receptor PYR/PYL/RCAR family by *S*-nitrosylation or tyrosine nitration at cysteine residues (Castillo et al., 2015). Tyrosine nitration under conditions in which NO is produced was proposed as a rapid mechanism limiting ABA signaling (Castillo et al., 2015). On the other hand, ABA signaling in guard cells includes H_2_O_2_ and NO production, which seems to be necessary for the ABA-induced stomata closure (Castillo et al., 2015). Thus, NO appears as a multifaceted plant growth regulator mediating direct modifications of key components in several hormone signaling pathways.

The ability of the root to grow toward higher water potential (hydrotropism) is an important physiological adaptation trait of plants to drought. The recent findings of Chang et al. (2019) have uncovered the importance of type-A ARRs in controlling root hydrotropism. ARR16 and ARR17 were found to be upregulated at the side of the root tip that “faces” lower water potential, resulting in increased cell division in the meristem zone and consequent bending of the roots toward the opposite side – in the direction of higher water potential.

Furthermore, various types of abiotic stresses including cold, osmotic stress, salt, and drought differentially regulate the expression of several individual MSP components (Argueso et al., 2009). As mentioned above, some of these regulations can be attributed to stress-induced ABA, however, the molecular mechanisms behind most of these regulations remain to be clarified.

### Ethylene and ABA Crosstalk During Osmotic Stress

Ethylene signaling too affects salt and osmotic stress responses. The receptors ETR1 and EIN4 inhibit, and ETR2 stimulates, seed germination under salt stress (Zhao and Schaller, 2004; Wilson et al., 2014b). It has been demonstrated that seeds of the loss-of-function *etr1-6* and *etr1-7* mutants have a higher germination rate than wild-type seeds in the dark or following FR illumination (that inhibits seed germination in the WT) indicating a negative role for ETR1 in the control of germination (Wilson et al., 2014a). The authors suggest that ETR1 may genetically interact with phyA and phyB to govern germination and growth under various light conditions (Wilson et al., 2014a; Figure 3). The ETR1 receptor may modulate ABA sensitivity even when ethylene does not bind to this receptor (Wilson et al., 2014a,b). ETR1 and ETR2 indirectly affect the expression of ABA signaling genes, without a requirement for canonical ethylene signaling (Bakshi et al., 2015, 2018). These findings show that ETR1 and ETR2 act independently of ethylene signaling to affect seed germination under salt stress by interacting with ABA signaling (Wilson et al., 2014b). Another crosstalk between ABA and ethylene was associated with TOR protein kinase (Dong et al., 2015). Inhibition of TOR kinase function led to increased expression of ethylene signaling components such ethylene response factors and ethylene responsive element-binding factors while down-regulating type-A response regulators (Dong et al., 2015). In line with that, TOR protein kinase has been shown to act as a central regulator of cell growth by integrating nutrient, energy, and growth factors in photosynthetic organisms (Schepetilnikov and Ryabova, 2018; Jamsheer et al., 2019).

Early osmotic stress response is integrated by ABA signaling through the hyperosmotic activation of RAF-like mitogen-activated protein kinases (MAPKKKs) that further activate downstream ABA-unresponsive and ABA-activated SnRK2s (Katsuta et al., 2020; Lin et al., 2020; Lozano-Juste et al., 2020; Soma et al., 2020). Fast response to osmotic stress is, however, also mediated by ABA-unresponsive subclass I and III SnRK2s independently to ABA (Boudsocq et al., 2007).

### MSP-Mediated Responses to Ambient Temperature Changes

Temperature is another fundamental environmental factor, with 10–30°C being optimum for the growth of most higher plants (Źróbek-Sokolnik, 2012). Temperatures below and above this optimum range are referred to as cold or heat stress, respectively (Larkindale et al., 2007; Miura and Furumoto, 2013). Both extremes may have fatal consequences for the plant (for details see, e.g., Xiong et al., 2002; Sung et al., 2003; Kaplan et al., 2004; Ahuja et al., 2010; Johnová et al., 2016).

In a response to adverse ambient temperatures, plants reprogram their transcriptome, metabolome, and proteome as well as their hormonal status to adapt their growth to the unfavorable temperature conditions (Stavang et al., 2009; Mittler et al., 2012). Cytokinin content is generally reduced by cold shock, which seems to be related to the reallocation of energy resources from growth toward defense. Prolonged cold treatment (for more than 3–7 days) is accompanied by plant acclimation (e.g., in winter wheat cultivars), associated with elevation of cytokinin content (Kosová et al., 2012). This finding is in line with the positive effect of exogenous cytokinins on freezing tolerance of *Arabidopsis* (Jeon et al., 2010; Maruyama et al., 2014). Thus, the role of cytokinins in cold responses and freezing tolerance seems to depend on the severity and phase of the stress response. Additionally, environmental stimuli such as temperature and water stress alter the phosphorylation status of MSP components partly independent of hormonal status suggesting that histidine kinases (and/or thermo-responsive phytochromes, see below) function as abiotic stress sensors, as shown for AHK1 (Tran et al., 2007; Jeon et al., 2010; O’Brien and Benková, 2013).

Even if enhanced freezing tolerance of *ahk2 ahk3* and *ahk3 ahk4* double mutants seems to be in accordance with the decreased cytokinin content under cold stress, it may be related, at least partially, to their low growth rate (Jeon et al., 2010). Cytokinin-independent (but nevertheless requiring functional cytokinin-responsive AHKs) upregulation of genes encoding the type-A response regulators ARR5, ARR6, ARR7, and ARR15 was reported as an early cold stress response (Jeon et al., 2010). Interestingly, the enhanced freezing tolerance observed in *arr7* and *ahk2 ahk3*, *ahk3 ahk4* mutants seems to act independently of the C-REPEAT-BINDING FACTOR/DRE-BINDING FACTORs (CBF/DREBs)-mediated cold acclimation pathway. Based on the hypersensitive response of *ahk2, ahk3*, and *arr7* mutants, as well as the insensitivity of *ARR7* overexpression line to ABA, the authors propose that the MSP-mediated cold response is mediated via negative regulation of ABA signaling by AHK/ARRs, thus following the general scheme of negative cytokinin/ABA interaction as in the drought response described above (Figure 1). Moreover, ARR1 was shown to function as a positive factor for cold signaling, acting downstream of AHK2, AHK3, and AHPs (Jeon and Kim, 2013; Figure 3). Interestingly, the majority of cold-responsive genes that are differentially regulated in *ahk2 ahk3* compared to WT, do not seem to be regulated via CBF3 or ARR1, suggesting the existence of yet unidentified (either direct or indirect) regulatory mechanisms, mediating the AHK2/3-dependent cold response (Jeon and Kim, 2013).

The CRF signaling branch of MSP is also involved in the response to cold stress, but also to heat, salt and oxidative stress, as well as elevated hydrogen peroxide content. All these stresses stimulate the activity of the CRF6 promoter. Overexpression of *CRF6* diminished oxidative stress impact, improving photosynthesis and stimulating root growth in *Arabidopsis* and tomato plants (Inzé et al., 2012; Zwack et al., 2013, 2016; Gupta and Rashotte, 2014). Other CRFs were also observed to be regulated by cold, enabling freezing tolerance by either inducing CRF4 or altering lateral root development in response to low temperature through CRF2 and CRF3 (Shi et al., 2014; Zwack and Rashotte, 2015; Jeon et al., 2016).

### Light-Related Stress Responses Through Multistep Phosphorelay

Light irradiance is an essential environmental factor influencing a wide range of physiological and growth aspects of the plant, and is tightly linked to plant hormonal status. An intimate link between light and cytokinins has been recognized for a long time now (reviewed in Cortleven and Schmülling, 2015; Zdarska et al., 2015). However, light can also act as a stressor, affecting both abiotic and biotic stress responses in plants (Roeber et al., 2020).

Cytokinins were shown to play an important role in photoinhibition induced by high dose irradiation. AHK2 and AHK3 and their downstream targets ARR1 and ARR12 were suggested to mediate cytokinin-dependent protection of the photosynthetic apparatus during high light stress (Cortleven et al., 2014; Cortleven and Schmülling, 2015). Cytokinins (through AHK3) were also found to modulate a novel type of stress called photoperiod stress, identified in plants with reduced endogenous cytokinin content or disturbed cytokinin signaling, and in circadian clock mutants (Nitschke et al., 2017). Photoperiod stress occurs due to abrupt changes in the alternation of light and dark periods, inducing symptoms of stress in *Arabidopsis* plants, including JA-dependent cell death and downregulation of the key circadian clock regulators CCA1 and LATE ELONGATED HYPOCOTYL [(LHY), (Figure 3; Nitschke et al., 2016, 2017). Recently it was found that the root-derived cytokinin *trans*-zeatin, acting via AHK2 and AHK3 in cooperation with AHK5, and the downstream type-B ARRs ARR2, ARR10, and ARR12, has a protective role against photoperiod stress, associated with an oxidative burst-like response (Abuelsoud et al., 2020).

Metaphorically, sensor kinases serve as guards at the gate to the cell or nucleus, enabling the perception of various environmental signals. Besides HKs, acting as an integral part of canonical MSP signaling, other sensor kinases integrated into MSP signaling are phytochromes (see above, Figure 1). Besides their major role in the light perception, phytochromes are also involved in osmotic-stress (Balestrasse et al., 2008) and drought responses (Kidokoro et al., 2009; D’Amico-Damião et al., 2015) (Figure 2). For instance, phyB was reported to positively affect drought tolerance in Arabidopsis (González et al., 2012). However, in other studies, phytochrome mutants exhibited improved drought tolerance (Liu et al., 2012), altered responses to high temperature (Njimona et al., 2014), and sensitivity to prolonged UV-B light radiation (Boccalandro et al., 2001; Rusaczonek et al., 2015). phys are necessary also for effective cold acclimation (Crosatti et al., 1999). phyB is stabilized at low temperature by the main cold-inducible TFs CBFs (Jiang et al., 2020). As phyB promotes degradation of PIF1, 4, and 5, which (especially PIF4) repress CBF genes at cold stress (Lee and Thomashow, 2012), phyB enhances freezing tolerance. Positive effect on freezing tolerance was found also in the case of phyA, especially at low R:FR ratio (Wang et al., 2016).

Importantly, phyB and its downstream signaling partner PIF7 were found to act also as thermosensors (Jung et al., 2016; Legris et al., 2016; Chung et al., 2020). PIF7 protein abundance was shown to accumulate in response to warm temperature further stimulating the expression of genes involved in thermomorphogenesis such as the auxin biosynthetic gene YUCCA8 (Chung et al., 2020). Interestingly, secondary structure of *PIF7* mRNA forms an RNA hairpin within the 5′UTR region which changes its conformation at higher temperature and stimulating the *PIN7* mRNA translation. This unique temperature-controlled mechanism of protein translation was identified also for WRKY22 and heat-shock regulator HSFA2 suggesting that as a conserved mechanism used by plants as a fast response to sudden temperature changes (Chung et al., 2020). Another rapid temperature-regulated mechanism was identified in case of phyB itself. phyB was postulated to be a temperature sensor in plants through its temperature-dependent reversion from the active Pfr state to the inactive Pr state, which affects its association with the promoters of key target genes in a temperature-dependent manner (Jung et al., 2016; Legris et al., 2016). Here, both temperature-dependent transcriptional regulation of *PIF4* together and phyB-mediated post-transcriptional regulation of PIF4 ability to activate transcription of target genes was proposed (Jung et al., 2016). The active Pfr form of phyB was shown to be stabilized by ARR4 *in planta*, which was confirmed by increased hypersensitivity to red light in *ARR4* overexpressing transgenic lines (Sweere et al., 2001; Fankhauser, 2002; see above). ARR3 and ARR4 seem to control both length of the period and its phase (Salomé et al., 2006). The loss of ARR4 and its closest homolog ARR3, prolongs the circadian cycle period even in the absence of light, suggesting that ARR3 and ARR4 control the circadian period independently of active phyB through an as yet unknown mechanism. In white light, *arr3* and *arr4* mutants show a leading phase (i.e., an earlier peak in the expression of genes that oscillate with the circadian rhythm) similar to *phyB* mutants, demonstrating that circadian light input is modulated by the interaction of phyB with ARR3 and ARR4. Considering the absence of any correlation between sensitivity to cytokinins in higher-order arr mutants and the period phenotype, as well as the distinct (concentration-dependent) effects of exogenous cytokinins, ARR3 and ARR4 seem to control both the length of the period (independent of phyB) and the phase of the period (through phyB) independently of cytokinins (Salomé et al., 2006). Since phyB acts as a light and temperature sensor and type-A *ARRs* expression responds to temperature fluctuations independently of cytokinin levels (see above), we may say that type-A ARRs could represent an intersection for light and temperature signaling to fine-tune photomorphogenesis under adverse environmental conditions (Figure 3).

Considering that stress responses are also modulated by cryptochromes (D’Amico-Damião et al., 2015), further research should be focused on the interconnection between blue light perception and MSP. Data about blue light – MSP crosstalk in abiotic stress responses is rather rudimentary. One of the potential mechanisms of blue light-cytokinin crosstalk might be mutual regulation of HY5 protein levels, as described above (Gangappa and Botto, 2016).

## Conclusion and Future Outlines

In our opinion one of the most critical roles of plant hormones is to act as a “universal” transducer between a constantly changing environment and control of the immediate physiological and developmental status of the plant. This transduction is then manifested as the final growth and developmental response. Multistep phosphorelay seems to be an ideal mechanism, allowing for the integration of multiple signaling inputs into a molecular response, characterized by quantitative and readily tunable changes in the transcriptional profile. In the light of several recent findings, implicating cytokinin-regulated MSP signaling (through type-B ARRs) in the control over context-dependent chromatin accessibility (Potter et al., 2018), the well-documented (cytokinin-modulated) role of ethylene in histone acetylation and ABA-dependent regulation of chromatin-remodeling complexes (see above), we can venture to say that epigenetic regulations form part of the repertoire of transcription regulatory tools available to MSP (and interconnected signaling pathways), possibly mediating environmental “memory” used by the plants in their adaptation to short- and medium-term environmental fluctuations. In line with this, Samsonová et al. (2020) showed that cytokinins occupy a prominent position among other hormones as unique ecotype identifiers. A positive correlation between mean ABA content and ABA/cytokinin ratio in the shoots with mean temperature at the site of origin is in line with existing data on the involvement of cytokinin signaling in the cold response (see above). This then means that control over cytokinin metabolism is one of the mechanisms involved in long-term adaptation. Also, the large variability in the ABA/cytokinin ratios observed among the ecotypes in both shoots and roots suggests hormonal plasticity, which might contribute to and/or determine stress responses in particular geographical regions (Samsonová et al., 2020).

The conclusions and implications we summarize above point toward a number of important questions. How is the combination of individual inputs translated into the final MSP signaling output? How is signaling fidelity maintained? In other words, how does the plant recognize and discern the particular origin of the signal, given that a number of sensory kinases recognizing the different type of signals result in phosphorylation of AHPs, and act as a signaling hub within the pathway? Is there any variability in the sensitivity of MSP signaling to individual types of MSP inputs associated with ecotype-specific adaptation to the site of its origin? And finally, what are the mechanisms acting downstream of MSP signaling in the stress response, either independently or in concert with other signaling pathways?

Future studies aimed at potential modification of the MSP signaling output at the level of the regulated genes and characterization of the underlying molecular mechanisms will be necessary to answer some of these questions. Studying the existing pool of natural genetic variability in MSP signaling affecting the responsiveness of the pathway to various inputs might provide important evidence on the potential role of MSP signaling and its downstream targets. They would also go a long way in shedding light on the ability of plants to successfully face environmental stresses which are after all an everyday reality of plant life. In combination with tools for targeted genome editing of plant genomes that have become available recently (Jinek et al., 2012; Anzalone et al., 2019), this type of knowledge will be a powerful enabler for rapid and targeted introduction of valuable alleles into the elite varieties being used in contemporary crop breeding programs.

## Author Contributions

JS, KN, and JH performed the literature search. KN and JS created the figures. JS, KN, RV, and JH wrote the manuscript. All authors contributed to the article and approved the submitted version.

## Conflict of Interest

The authors declare that the research was conducted in the absence of any commercial or financial relationships that could be construed as a potential conflict of interest.
